# The AI Act and the MDR post-market requirements for semiautonomous AI SaMD: a radiology case study in prostate cancer

**DOI:** 10.1007/s00261-026-05434-z

**Published:** 2026-02-20

**Authors:** Shakila Shojaei, Derya Yakar, Nynke Vellinga, Vilma Bozgo, Thomas Kwee, Henkjan Huisman, Jeanne Pia Mifsud Bonnici

**Affiliations:** 1 Department of Radiology, University Medical Center Groningen, Groningen, Netherlands; 2 Security, Technology and e-Privacy Research Group (STeP), University of Groningen, Groningen, Netherlands; 3 Diagnostic Image Analysis Group, Radboud University Medical Center, Nijmegen, Netherlands

**Keywords:** Artificial intelligence, Postmarketing, Prostatic neoplasms, Medical Device Regulation, Magnetic resonance imaging

## Abstract

**Purpose:**

To clarify overlapping post-market obligations under the EU Artificial Intelligence Act (AIA) and EU Medical Device Regulation (MDR) for high-risk artificial intelligence (AI) Software as a Medical Device (SaMD), and to map the regulatory landscape for manufacturers, healthcare providers, AI providers, and AI deployers.

**Methods:**

We conducted a qualitative doctrinal legal analysis of post-market provisions in the AIA and MDR, using a case study of a high-risk Class III AI SaMD for prostate cancer radiology. No empirical clinical or performance data were collected. The analysis focused on key stakeholders, including device manufacturers and deployers (e.g., healthcare providers). We sought to identify (1) convergence, where both regulations impose overlapping or complementary requirements, and (2) divergence, where obligations are addressed by only one regulation, revealing potential regulatory gaps.

**Results:**

We organized the extracted post-market obligations into ten categories. Overall, both regulations place increasing emphasis on lifecycle traceability and continuous monitoring. We identified convergence in areas such as documentation and performance monitoring, while divergences emerged in domains like human oversight (in the AIA) and reporting non-serious patterns (in the MDR). We also identified gaps in regulatory guidance, particularly regarding system updates, human oversight, and the evolving responsibilities of healthcare providers.

**Conclusion:**

The AIA and MDR share common ground in some post-market areas but also diverge in key responsibilities. To ensure safe and effective use of high-risk AI in healthcare, clearer coordination between the two frameworks is needed, especially in areas such as human oversight and system modification, where current guidance remains limited.

**Supplementary Information:**

The online version contains supplementary material available at 10.1007/s00261-026-05434-z.

## Introduction

Healthcare is under mounting pressure from rising costs, surging demand, and the imperative for high-quality care [1]. In radiology, and especially in prostate magnetic resonance imaging (MRI), this escalating demand is intensifying capacity constraints as specialist availability remains limited, creating a clear need for more efficient diagnostic pathways [2, 3]. Artificial intelligence (AI) promises gains in diagnostic accuracy and efficiency [4, 5]; for example, prostate MRI triage using AI can reduce radiologist workload by filtering straightforward non-cancer cases, yielding a 12.5% reduction [6]. However, integrating AI into clinical workflows introduces operational and governance challenges [7]. These challenges affect stakeholders, including AI manufacturers, healthcare providers, and clinicians who deploy and use these systems in practice.

**Fig. 1:**
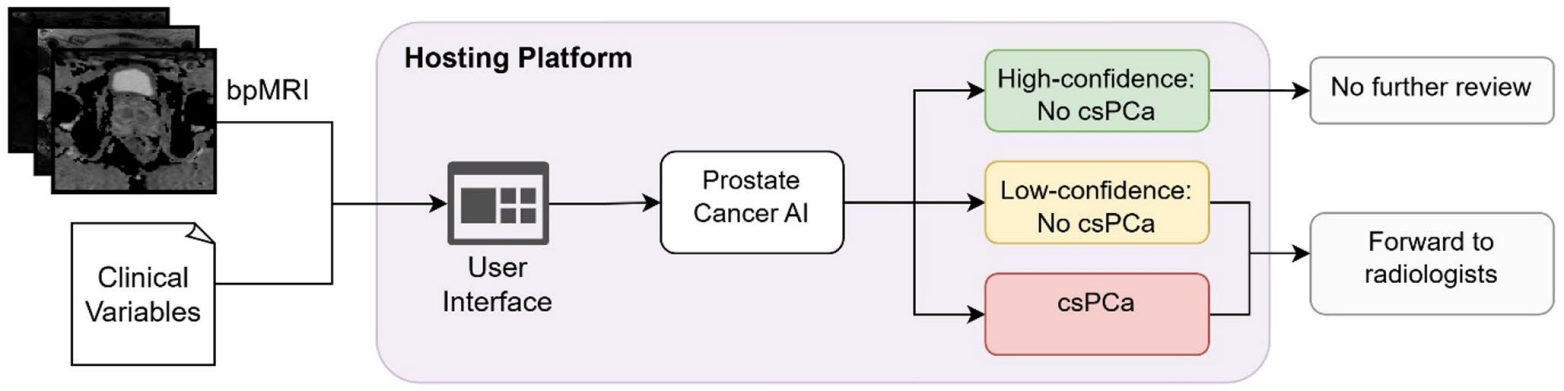
Example workflow for AI-assisted prostate MRI triage in a semiautonomous setting. Biparametric MRI (bpMRI) scans and relevant clinical variables are processed by a prostate MRI triage model. This model is integrated into a hosting platform via a user interface. Arrows indicate the flow direction from left to right. The model stratifies cases into high-confidence negatives (automatically triaged with AI output as final diagnosis), low-confidence negatives, and likely clinically significant prostate cancer (csPCa). Cases not confidently excluded are forwarded to radiologists for further review. MRI images in this illustration are reproduced from the PI–CAI Public Training and Development Dataset [12], and are used with permission from the rights holder (no changes were made to the original images)

This study addresses a targeted part of these governance challenges by examining how the EU Artificial Intelligence Act (AIA) [8] and the EU Medical Device Regulation (MDR) [9] jointly shape and allocate post-market oversight responsibilities, with particular attention to healthcare providers deploying prostate MRI AI systems. We ground the analysis in a prostate cancer triage case study in a semiautonomous setting (Fig. 1). The system detects clinically significant prostate cancer (csPCa) using biparametric magnetic resonance imaging (bpMRI) and relevant clinical variables. As a representative example, we refer to the Prostate Imaging–Cancer Artificial Intelligence (PI–CAI) system developed for an international benchmarking challenge [10]. In our setup, it operates via a hosting platform such as Grand Challenge that receives input data and returns AI-driven diagnostic outputs [11].

In the European Union (EU), these AI-enabled systems intended for a medical purpose are already regulated under the MDR, which mandates safety, performance, and clinical evaluation [9]. With the adoption of the AIA, they must also satisfy AI-specific obligations [8] that extend responsibilities beyond manufacturers to healthcare providers, further complicating adoption pathways [13]. Across these two regulations, core obligations such as risk management, technical documentation, quality management systems (QMS), and post-market oversight are mandated. This overlap may lead to duplication, ambiguity, and conflict [14, 15], thereby imposing dual compliance burdens on manufacturers and healthcare providers. A recent qualitative study with manufacturers and regulators underscored the need for clearer guidance and harmonized standards to bridge this gap [16]. In this direction, the Medical Device Coordination Group (MDCG) published a guidance document in June 2025 on the interplay between the AIA and MDR across key regulatory areas, including post-market requirements [17]. However, its broad scope leaves a need for more detailed case studies. Several studies have further explored the alignment between these two regulations. Van Kolfschooten and van Oirschot [18] provided a legal overview of the AIA’s impact on healthcare stakeholders. Meanwhile, van Leeuwen et al. [19] identified additional responsibilities for deployers of high-risk AI systems beyond the MDR mandates. The European Society of Radiology (ESR) issued radiology-specific recommendations revealing implementation gaps [20]. However, these studies remain high-level, leaving a clear gap in applied analyses of how these frameworks operate in practice.

In our case study, the system qualifies as high-risk AI under the AIA and Class III Software as a Medical Device (SaMD) under the MDR. This creates a high-stakes context for examining post-market obligations. The post-market phase is crucial for safeguarding patient safety, especially since AI performance may shift post-deployment, requiring robust ongoing oversight [21]. By mapping post-market obligations for AIA providers, deployers, and their MDR counterparts in this prostate MRI triage workflow, we clarify how each framework allocates responsibility and lay the groundwork for further radiology research on AI implementation and monitoring. The combination of clinical relevance and regulatory complexity highlights the study’s importance, as it identifies domain-specific challenges and supports safe and effective AI adoption in healthcare.

For clarity, SaMD refers to standalone software intended for a medical purpose under the MDR definition. This includes AI SaMD, where the software’s medical functionality is primarily AI-driven. For example, it may use AI to analyze clinical data or medical images and to support or generate outputs for disease detection, diagnosis, triage, or clinical decision support. Under the AIA, the provider typically corresponds to the MDR manufacturer, while the deployer refers to the healthcare provider or institution that implements and uses the system. The post-market stage refers to the period after the system is placed on the market or put into service and incorporated into real-world clinical workflows. During this stage, ongoing oversight is required to detect emerging risks, performance drift, or safety issues and to initiate corrective or preventive actions. Although both frameworks impose post-market obligations, for the purposes of this analysis, we maintain distinct terminology by using post-market monitoring (PMM) under the AIA and post-market surveillance (PMS) under the MDR to preserve conceptual clarity.

## Methodology: extracting and grouping post-market obligations

We conducted a qualitative doctrinal legal analysis of the AIA and MDR as an interdisciplinary team of AI specialists, radiologists, and technology law experts. The process followed four main steps: (1) assessing the applicability of both regulations to the system under study; (2) determining classification under each; (3) extracting the legal obligations related to the post-market stage; and (4) organizing overlapping and distinct requirements into unified obligation groups. The AI and legal team met regularly (twice per month over seven months) to align the extraction approach, resolve ambiguities, and agree on how to group and describe requirements. Radiologists then reviewed the outputs for clinical relevance and clarity, and four joint alignment meetings were held to confirm interpretations.

We used the English versions of the AIA (OJ L, 2024/1689, 12.7.2024) [8] and the MDR (OJ L 117, 5.5.2017) [9]. Our analysis is limited to the binding EU-level requirements in these two regulations that apply to the specific case study system described in this paper. We did not conduct a detailed analysis of other EU legal regimes referenced in the AIA/MDR, such as the General Data Protection Regulation (Regulation (EU) 2016/679) [22] or the Regulation on market surveillance (Regulation (EU) 2019/1020) [23]. We also did not assess national implementing laws, national guidance, or country-specific enforcement practices. Of all MDCG guidance documents, we consulted only MDCG 2021-24 [24], solely to support MDR device classification.

**Fig. 2:**
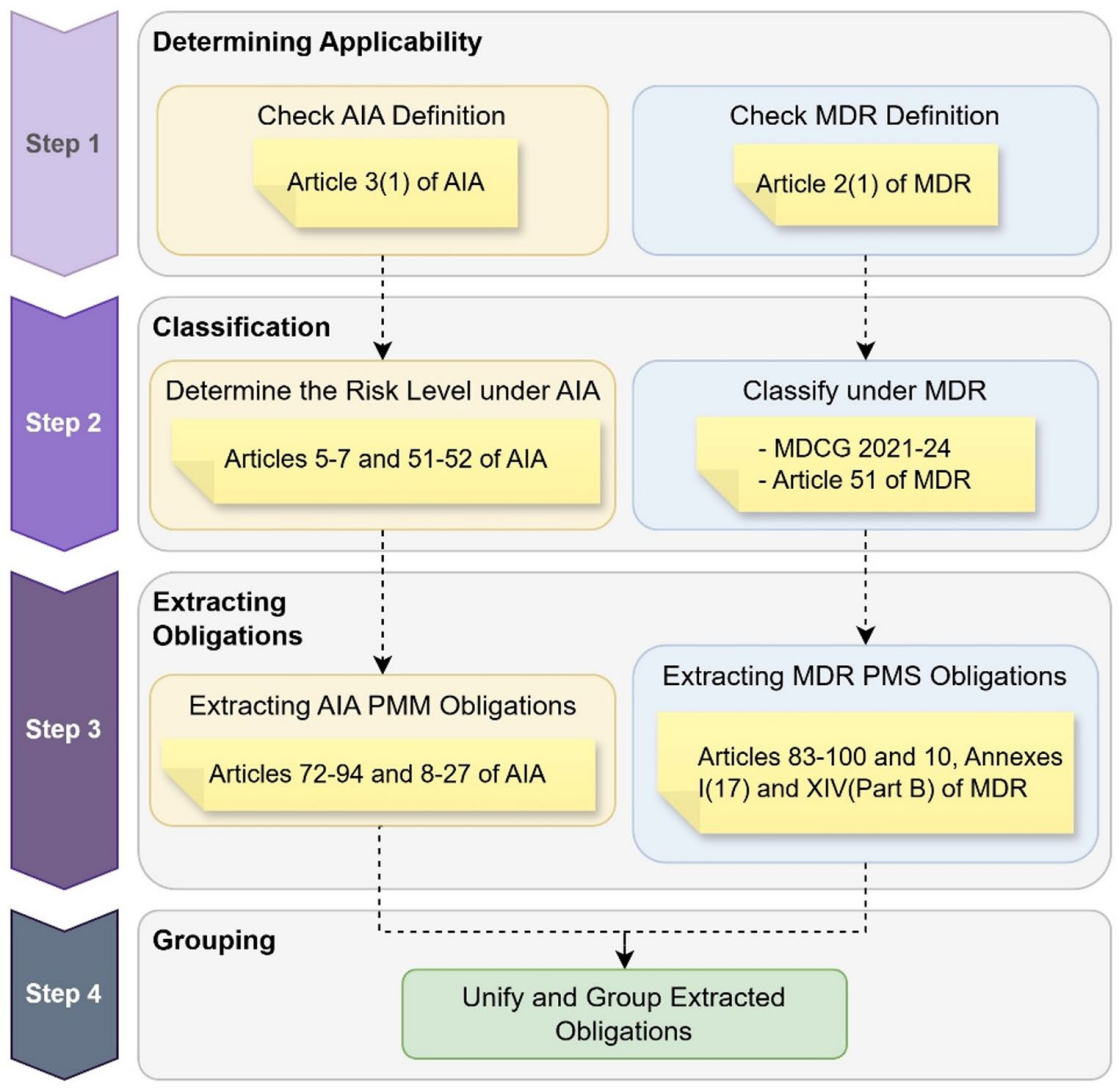
Overview of the steps for extracting and grouping post-market stage obligations under the AIA (left) and MDR (right), with arrows indicating progression to the next step. In Step 1, the applicability of each regulation is assessed via definition articles. In Step 2, the AI SaMD is classified under both AIA and MDR. In Step 3, distinct obligations are extracted by reviewing relevant articles of AIA (for AI providers/deployers) and MDR (for medical device manufacturers/healthcare providers). In Step 4, final obligation groups are formed

Both the AIA and MDR include specific sections that set out activities and duties for the post-market stage. We used these post-market sections as the primary basis for extracting obligations. To ensure completeness, we also included a limited set of broader provisions that directly shape post-market oversight for the system under study, such as general obligations for high-risk AI systems under the AIA and selected general manufacturer obligations under the MDR. We extracted obligations at the article level (and, where needed, paragraph/subparagraph level) using an extraction template that we developed at the start of the analysis and refined iteratively during the process. The template captured the obligation holder, a plain-language obligation title (required action), the applicable device type or class where specified, and relevant cross-references. We then grouped obligations through iterative text-based comparison, distinguishing related versus distinct requirements across the two regulations and refining the groupings through interdisciplinary consensus. Figure 2 summarizes the methodology and regulatory articles reviewed. Each step is described in detail below.

### Determining applicability

First, we reviewed the definition articles of each regulation. Article 3(1) of the AIA defines an *AI system*, and Article 2(1) of the MDR defines a *medical device*. This confirms that both frameworks apply, and their obligations are relevant.

### Classification

The AIA categorizes AI systems as prohibited, high-risk, limited-risk, or minimal-risk. Classification begins by excluding prohibited uses (Article 5). It then assesses high-risk AI criteria (Articles 6–7). For general-purpose systems, additional obligations apply (Articles 51–52). In parallel, the MDR classifies medical devices into Classes I, IIa, IIb, and III based on intended purpose and risk level (Article 51, Annex VIII), with MDCG 2021-24 providing further guidance [24]. However, under the AIA, systems used in products falling under EU harmonization laws (Annex I), such as the MDR, and requiring third-party conformity assessment (e.g., Class IIa, IIb, or III devices under the MDR) are automatically classified as high-risk (Article 6(1)) [20].

### Extracting obligations

This step identifies the post-market obligations under the AIA and MDR for AI providers, AI deployers, medical device manufacturers, and healthcare providers. Under the AIA, key PMM obligations are outlined in Chapter IX (Articles 72–94), with additional provisions varying by risk classification. For example, since our case study is likely high-risk, the analysis also covers Chapter III, Sections 2 and 3 (Articles 8–27), which set core obligations for high-risk AI. Similarly, PMS obligations under the MDR are primarily outlined in Chapter VII (Articles 83–100). Annex XIV further specifies Post-Market Clinical Follow-up (PMCF) as a continuous process of collecting real-world clinical data to confirm safety and performance and to identify emerging risks. Additionally, Article 10 outlines manufacturers’ general obligations, including PMS alongside other responsibilities. Finally, given the software characteristics of the case study, Section 17 of Annex I, which addresses electronic programmable systems and standalone software, is also considered.

### Grouping and unifying obligations

After extracting the relevant obligations from the AIA and MDR, we organized them into cohesive thematic groups. These groups were then compared across both regulations to identify areas of convergence and divergence.

## Results: applying methodology to the case study

The case study examined in this analysis qualifies as a high-risk AI system under the AIA and a Class III medical device under the MDR. Detailed reasoning for regulatory applicability and classification (Steps 1 and 2) is provided in Online Resource 1 (Supplementary Text 1). Post-market obligations for providers and deployers under the AIA are summarized in Online Resource 2 (Supplementary Table 1); corresponding obligations for manufacturers and healthcare providers under the MDR are outlined in Online Resource 3 (Supplementary Table 2). Online Resource 4 (Supplementary Table 3) lists the articles reviewed but excluded from the final analysis, with reasons for exclusion.

We identified post-market obligations of our target obligation holders under both regulations and organized them into ten thematic groups. Table 1 presents these groups with the corresponding AIA and MDR articles, highlighting areas of convergence and divergence. Table 2 provides a condensed overview of the obligations within each group and identifies the main obligation holder among each of the four roles (AI provider, AI deployer, manufacturer, healthcare provider/organization). An expanded version of Table 2 is provided in Online Resource 5 (Supplementary Table 4). In this analysis, AI providers correspond to manufacturers, and AI deployers correspond to healthcare providers. Although the text uses the terms *AI provider* and *AI deployer*, our analysis primarily refers to *manufacturers* and *healthcare providers*. The following section provides a detailed analysis of each obligation group.

**Table 1:** Ten post-market obligation groups for manufacturers and deployers in an AI-assisted prostate cancer triage case study representing a Class III high-risk AI SaMD

Obligation Group	AIA Articles	MDR Articles	Convergence–Divergence (AIA «« « ● » »» MDR) ^a^
1. Planning a post-market oversight system	9(2)(c), 17(1)(h), 72 Annex IV(9)	83(1–3), 84, 10(9)(f), 10(9)(i), 10(9)(m), 10(10), Annex I Chapter I(3)(e), Annex XIV(5–8)	●
2. Trend reporting of non-serious incidents	N/A	88	**»»**
3. Serious incident reporting and advance notification of corrective actions	17(1)(i), 26(6), 73(1–5), 73(10)	10(9)(k), 10(13), 83(4), 87(1–9), 87(11)	**»**
4. Investigation and risk evaluation after serious incident reporting	20(2), 21(1), 73(6)	89(1), 89(3), 89(5)	●
5. Corrective actions and safety notices after serious incidents	16(j), 20	10(9)(l), 10(12), 83(4), 89(8)	**»**
6. Addressing suspected risks and non-compliance outside incident reporting pathways	16(j-K),17(1)(a), 20(1), 21	10(9)(a), 10(12), 94, 95(1), 95(3), 97(1)	**»**
7. Market surveillance readiness across the product life cycle	21, 74(1), 74(5), 74(12–13)	10(8), 10(14), 86, 93(3)	**»**
8. Maintaining ongoing documentation and logs	11(1), 12(1), 12(2)(a-c), 16(e), 18(1), 19(1), 26(6)	10(4), 10(8)	**«**
9. Human oversight	14(1–4), 26(2), 26(5)	Partially 17(1) of Annex I	**««**
10. Requirements for safe and transparent AI technology	10(1–5), 15(1), 15(4–5), 26(4), 86	Annex I (17.1), Annex I (17.2), Annex I (17.4)	**««**

Each group is mapped to the corresponding AIA and MDR articles, and the final column summarizes convergence (overlapping/complementary obligations) versus divergence (regulation-specific obligations)

^a^ «« strongly AIA-leaning, « slightly AIA-leaning, ● broadly overlapping (balanced), » slightly MDR-leaning, »» strongly MDR-leaning

**Table 2:** Overview of post-market obligation groups and primary obligation holders under the AIA and MDR in an AI-assisted prostate cancer triage case study representing a Class III high-risk AI SaMD

Obligation Group	Obligation	Obligation Holder^a^			
		AI Provider (AIA)	AI Deployer (AIA)	Manufacturer (MDR)	Healthcare Provider or Organization (MDR)
1. Planning a post-market oversight system	Embedding a Post-Market Monitoring (PMM)/Post-Market Surveillance (PMS) System within the Quality Management System (QMS)	X		X	
	Post-Market Data Collection and Use	X		X	
	Structured Use of PMM/PMS Data for Continuous Improvement	X		X	
	Structured and Documented PMM/PMS Plan	X		X	
	Alignment of PMM with Existing Sectoral Monitoring Systems	X			
2. Trend reporting of non-serious incidents	Detecting and Reporting Statistically Significant Increases in Non-Serious Incidents or Expected Side Effects			X	
	Integrating Trend Monitoring Methodology in the PMS Plan			X	
	Implementing Competent Authority-Mandated Measures after Trend Report Review			X	
3. Serious incident reporting and advance notification of corrective actions	Reporting Serious Incidents and Field Safety Corrective Actions (FSCAs) to Authorities	X	*	X	*
	Ensuring Reporting within Severity-Based Timeframes	X	*	X	
	Submitting Incomplete Reports When Necessary	X	*	X	
	Reporting Uncertain Incidents within the Applicable Timeline			X	
	Replacing Individual Incident Reports with Periodic Summaries			X	
	Reporting Authority-Triggered Serious Incidents			X	
	Reporting PMS-Triggered Serious Incidents			X	
4. Investigation and risk evaluation after serious incident reporting	Initiating Investigation and Risk Assessment of Serious Incidents	X	*	X	
	Preserving System State and Cooperating During Investigations	X		X	
	Providing Documentation to Authorities for Post-Incident Assessment	X		X	
	Submitting the Final Investigation Report to Authorities	X		X	
5. Corrective actions and safety notices after serious incidents	Issuing and Distributing Field Safety Notices (FSNs) to Device Users			X	
	Implementing Corrective and Preventive Actions (CAPA) and Informing Authorities and Stakeholders	X		X	
6. Addressing suspected risks and non-compliance outside incident reporting pathways	Cooperating with the Competent Authority in Evaluations of Devices Suspected of Unacceptable Risk or Non-Compliance	X		X	
	Taking Corrective Actions for Devices Presenting an Unacceptable Risk	X		X	
	Correcting Non-Compliance That Does Not Pose an Unacceptable Risk			X	
	Demonstrating Compliance upon Request	X		X	
	Including a Regulatory Compliance Strategy in the QMS to Manage Modifications	X		X	
7. Market surveillance readiness across the product life cycle	Structured Safety Reporting Using the Periodic Safety Update Report (PSUR)			X	
	Applying Regulation (EU) 2019/1020 [23]	*	*	*	*
	Ensuring Access for Competent Authorities and Market Surveillance Authorities (MSAs) to Documentation, the Device, Code, Logs, and Datasets for Market Surveillance Conformity Checks	*	*	X	
8. Maintaining ongoing documentation and logs	Maintaining Technical Documentation	X		X	
	Retaining Compliance Documentation for the Specified Period	X		X	
	Keeping System Logs While the System Is Under Control	X	X		
	Ensuring Automatic Logging of Events	X			
	Logging Risk-Related Events and Substantial Changes	X			
	Logging Events for Post-Market Monitoring	X			
	Logging Events for Operational Monitoring by Deployers	X			
9. Human oversight	Designing and Developing AI Systems to Enable Effective Human Oversight During Use	X			
	Ensuring Oversight Reduces Risks	X			
	Aligning Oversight Measures with System Risk, Autonomy, and Context	X			
	Identifying and Implementing Human Oversight Measures	X	*		
	Ensuring Oversight Personnel Can Understand, Interpret, Override, and Interrupt the System	X			
	Assigning Competent and Supported Oversight Personnel		X		
	Monitoring System Operation and Reporting Risks or Incidents		X		
10. Requirements for safe and transparent AI technology	Providing Clear Explanations of AI-Based Decisions		X		
	Ensuring Accuracy, Robustness, Cybersecurity, and Consistent Performance Throughout the System Lifecycle	X		X	
	Ensuring Feedback Loops Are Not Influenced by Biased Outputs	X			
	Maintaining Cybersecurity Against Post-Deployment Attacks	X		X	
	Ensuring Input Data Are Relevant and Representative		X		
	Ensuring Data Quality and Governance	X			

^a^ “X” indicates the primary obligation holder; “*” indicates obligations that apply only where applicable (i.e., partially or in specific situations)

### Planning a post-market oversight system

A structured post-market oversight system is essential to detect issues early and ensure ongoing safety and performance after deployment. In high-risk settings such as healthcare, pre-market approval alone cannot guarantee long-term safety and performance. This is particularly relevant for AI systems, where real-world deployment may reveal failure modes and performance shifts that were not evident during pre-market evaluation [25, 26]. Both the AIA and MDR require such monitoring as part of a QMS tailored to device risk, purpose, and technology. Since our case study falls into the highest risk category (AIA high-risk, MDR Class III), comprehensive post-market oversight is critical. The AIA sets general obligations and enables alignment with sector-specific frameworks, such as the MDR, with a standardized template expected by February 2026. The MDR is more detailed, requiring manufacturers to gather real-world data, assess ongoing safety and performance, and update clinical evaluation, usability, and risk management. Manufacturers hold primary responsibility for the post-market system, while healthcare providers provide the real-world evidence on which it relies. MDR Article 83(3) requires post-market data to detect problems and support improvement, highlighting the central role of clinical workflows. In our case study, each interaction (confirming or overriding the AI output, flagging unusual cases, correcting heatmaps) should be recorded. For example, if a case triaged to the rule-out pathway is later escalated to radiologist review because new clinical information raises suspicion of csPCa, the original AI output, the escalation decision, and the reason for the escalation should be captured as post-market evidence. Therefore, when integrating the triage system into the diagnostic pathway, the workflow should identify roles interacting with the AI at each step and define their responsibilities, ensuring the collection of high-quality post-market data.

### Trend reporting of non-serious incidents

Tracking and reporting trends in non-serious incidents helps surface early safety signals before they escalate. Such signals often appear as minor incidents or expected side effects that, when increasing over time, may indicate broader safety or performance issues. While the AIA lacks a dedicated clause, the MDR requires manufacturers to monitor and report significant increases in such events, assess them using predefined PMS methods, and respond when thresholds are met or when authorities request action. Healthcare providers do not submit trend reports, but the data they produce is essential. In our prostate MRI triage case study, relevant trends could include a rising frequency of clearly positive advanced-stage cancers receiving low-confidence outputs, or an increasing number of cases in which the classification is correct, but the heatmap localization becomes less aligned with the lesion over time. Recording these patterns and communicating them to the manufacturer supports earlier detection and mitigation of emerging performance degradation that could affect triage safety.

### Serious incident reporting and advance notification of corrective actions

Serious incidents must be reported promptly, and planned corrective actions should be notified in advance through the manufacturer’s QMS processes. For AI SaMD settings, formal notifications ultimately go to the national competent authority for medical devices in the member state where the incident occurred. Under the AIA (Article 74(3)), the same sectoral authority performs market surveillance for MDR-covered products. Manufacturers remain the primary party responsible for submitting the serious incident report, while healthcare providers have a complementary duty to rapidly identify suspected incidents, preserve evidence, and immediately inform the provider and the relevant authority (or report directly if the provider cannot be reached). In our case study, any missed or delayed diagnosis of csPCa is an incident. For example, if several triaged cases are later found to have csPCa during discrepancy review, the site should flag this cluster as a suspected serious incident and report it, while users should be notified in advance of any corrective action that changes the workflow (e.g., threshold adjustments, model update, or temporarily disabling the rule-out pathway). Site-specific or subgroup (e.g., ethnicities) performance drops should also trigger reports for fairness concerns. Both frameworks require reporting immediately after establishing (or reasonably suspecting) a causal link and, in any event, within severity-based deadlines. Under the AIA, providers (or deployers, where applicable) must report within 2 days for widespread infringements or critical infrastructure disruptions, within 10 days for deaths, and within 15 days for other serious incidents. Under the MDR, manufacturers must report within 2 days for serious public health threats, 10 days for death or an unanticipated serious deterioration in a patient’s condition, and 15 days for other serious incidents. Initial reports may be submitted with incomplete information, provided they are updated promptly. The MDR also expects reporting even when there is uncertainty about reportability. When multiple similar incidents occur, for example, missed csPCa cases with a shared failure mode, the MDR may allow a summary report.

### Investigation and risk evaluation after serious incident reporting

Serious incident reporting must be followed by a documented investigation and risk evaluation before corrective measures are implemented. Ad hoc updates, recalls, or changes based on initial judgment should be avoided. The MDR requires manufacturers to document risk evaluation and determine necessary actions. The AIA also calls for prompt root-cause investigation with deployers, meaning that, in clinical use, healthcare providers support these investigations. In our case study, radiology teams should preserve evidence (MRI scans, AI outputs, heatmaps), document clinical impact (missed or delayed csPCa), and avoid local fixes during the investigation. Throughout the investigation, manufacturers and healthcare providers must cooperate with the relevant authority and provide the required documentation. Afterwards, in accordance with Article 89 of the MDR, the manufacturer must issue a final report documenting the investigation’s findings, while the AIA requires providers to notify the authorities of the serious incident and the corrective actions taken. An important consideration is that, under the AIA Article 74(4), for AI medical devices under the MDR, the AIA procedures in Articles 79–83 (including procedures for evaluating AI systems presenting a risk) do not apply where the MDR provides procedures with the same objective and an equivalent level of protection. In such cases, the relevant MDR procedures apply instead.

### Corrective actions and safety notices after serious incidents

After a serious incident is investigated and confirmed, manufacturers must implement corrective action without delay and notify relevant stakeholders. The response may involve corrective or preventive measures to restore conformity or, if this is not feasible, withdrawal or recall. Similar actions may also be required when general non-compliance is identified through routine monitoring. Under the MDR, after a Field Safety Corrective Action (FSCA) is taken, users must be promptly informed via a Field Safety Notice (FSN). The FSN details the action, the affected device or software versions, the risk, and the steps required from users. It is also submitted to the authority for comment, unless urgent, and made public (e.g., in the European database on medical devices). In our case study, healthcare providers should route FSNs to the relevant teams and implement them by updating or rolling back AI, restricting use if needed, and updating procedures accordingly. For example, if an FSN is issued because the rule-out pathway shows an increased rate of missed csPCa under a specific scanner protocol or acquisition setting, the site may need to temporarily disable the rule-out pathway for those configurations and route cases to radiologist review until the corrective update is implemented.

### Addressing suspected risks and non-compliance outside incident reporting pathways

Suspected unacceptable risk or non-compliance (not posing unacceptable risk) can arise without an incident report and must still be investigated, documented, and addressed. Such concerns may be raised through market surveillance activities, and manufacturers are expected to cooperate with the competent authority by providing the requested information and supporting evidence. Signals can originate from complaints, logs, routine monitoring, or the non-serious trends described in Group 2. If non-compliance or unacceptable risk is confirmed, manufacturers must act and inform stakeholders. While manufacturers are responsible, information from healthcare providers is critical for evaluation. In our case study, clinician inputs, such as agreeing, modifying, or rejecting dispositions, notes on unusual cases, and brief comments on heatmap localization and data quality, provide key evidence. Both frameworks also require the ability to demonstrate compliance on request and to manage system changes within the QMS. Because AI behavior can change after updates, healthcare providers should stay informed about changes, verify compatibility, and record the verification process. For example, when an update changes triage thresholds, the healthcare provider should record who approved the change, what checks were performed, and whether temporary safeguards (e.g., routing borderline cases to radiologists) were introduced.

### Market surveillance readiness across the product life cycle

Manufacturers and, where relevant, healthcare providers must be prepared for market surveillance throughout the product life cycle. This means granting authorities timely access to technical documentation, logs, datasets, and, when justified, source codes. The MDR also requires a Periodic Safety Update Report (PSUR), which summarizes PMS findings, clinical evidence, and usage estimates, including patient population and frequency of use. Maintaining the PSUR is the manufacturer’s responsibility; however, it should also include provider-supplied real-world evidence, such as the proportion of triaged cases, results of random checks performed under the site’s oversight procedure, scanner stability, key performance metrics, and records of incidents or near misses, along with corrective actions.

### Maintaining ongoing documentation and logs

Many post-market obligations (reporting, incident investigation, corrective actions, and market surveillance) rely on tracing system behavior over time, which requires robust documentation and logging. Both regulations require manufacturers to maintain technical documentation and provide it to authorities on request. In our case study, this documentation must be retained for a period of ten years. If a product falls under both regulations, the AIA allows a single documentation set for both frameworks. The AIA also requires lifecycle logging by manufacturers and deployers, with logs retained for a minimum of six months. The system must automatically generate logs throughout its operation, capturing risk indicators and substantial changes. In our case study, system logs should capture information such as AI decisions, confidence scores, model versions, scanning configurations, timestamps, user actions and overrides, misses and near misses, and clinician notes. The logs should also enable traceability over time by allowing reconstruction of how a given triage decision was produced and handled in the clinical workflow, thereby supporting ongoing post-market oversight.

### Human oversight

Human oversight ensures high-risk AI outputs are safe and overridable. The AIA requires providers and deployers to appoint qualified personnel and implement mechanisms to monitor performance, interpret outputs, and intervene when necessary. The AIA mandates four key oversight requirements: (1) integration into system design (e.g., pause or override controls, confidence indicators), (2) contribution to risk mitigation, (3) proportionality to risk and context, and (4) assignment of responsibilities to qualified individuals. In our case study, in addition to a conservative rule-out pathway that sends uncertain inputs to manual review, the platform must also allow users to override AI decisions and route cases to radiologists. For example, if image quality is poor or the output appears inconsistent with the scan, the user should escalate the case to a radiologist and document the reason. Also, as our system is semiautonomous, stricter local monitoring aligned with the prostate cancer workflow needs to be defined and implemented. Personnel with oversight duties must understand both the AI system and prostate MRI workflow to accurately interpret outputs and determine when to override. The MDR lacks a specific human oversight requirement. While Annex I, Section 17(1) calls for safety mechanisms that could imply override capabilities, it does not define oversight duties as the AIA does.

### Requirements for safe and transparent AI technology

The AIA requires providers and deployers to ensure AI technology is safe, transparent, and trustworthy. This includes maintaining accuracy, robustness, and cybersecurity throughout a product’s lifecycle, mirroring MDR demands for programmable medical devices (Annex I, Section 17). The AIA also expands safeguards against bias, enforces robust data governance, and requires justification for the use of sensitive data. Clinicians must provide relevant input data and support appropriate use in practice. For example, if key sequences are missing, corrupted, or affected by artifacts, the case should be flagged and routed to manual review rather than relying on the AI triage output. After software or model updates, the site should also verify the deployed model version and document basic checks showing that triage outputs remain consistent under the local MRI protocol. Clinicians should be able to explain the AI system’s role to affected individuals, including what the system does and does not do, and how AI-supported triage may influence the diagnostic pathway in prostate cancer.

## Discussion

This study analyzed post-market obligations of the MDR and AIA for a high-risk Class III AI SaMD in prostate cancer triage. By mapping AIA roles (providers and deployers) to MDR roles (manufacturers and healthcare providers), we identified areas of convergence (where both regulations impose overlapping or complementary responsibilities) and divergence (where obligations are addressed by only one framework). Our findings show that both frameworks emphasize traceability and continuous monitoring throughout the system lifecycle. The MDR provides a well-established framework for manufacturers focused on safety and surveillance. The AIA introduces additional requirements, including logging, human oversight, and more detailed provisions on system performance, robustness, and data governance. Importantly, as highlighted by Aboy et al., the AIA extends regulatory responsibility to include deployers, such as healthcare professionals and clinical staff [13]. This explicitly addresses a regulatory gap in the MDR, complicating responsibility mapping across these two frameworks.

Human oversight is a central but potentially ambiguous requirement under the AIA, and it can be interpreted as either verifying system outputs (e.g., confirming a detected lesion in our case study) or ensuring proper system functioning (e.g., storing and reviewing logs). Without standards for clinical use, oversight can reduce both efficiency and safety. For example, requiring expert review of every case would undermine the value of semiautonomous triage systems. A more practical approach is to align oversight intensity with the system’s intended level of autonomy, using safeguards that support selective escalation of uncertain cases rather than manual review of all outputs. In triage workflows, this means that routine review of every output may be unnecessary when safeguards are in place, and rule-out performance is demonstrated, particularly when sensitivity and negative predictive value are comparable to those of expert radiologists [27]. The system should also be subject to continuous monitoring and support timely intervention when behavior changes. At the same time, relying on clinician vigilance as the primary oversight mechanism may be unsustainable, as attention can erode under productivity pressures and routine AI use [28]. Shifting oversight to less experienced staff may simply move the workload and create new risks. Lack of defined qualifications and training can lead to inconsistent oversight practices [29]. Taken together, these considerations highlight the need for clearer, workflow-aligned standards for human oversight to support safe and efficient clinical integration of AI systems.

Our case study shows that post-market compliance increasingly relies on radiology departments. These departments are not only AI users but also generate evidence and perform ongoing monitoring in clinical practice. As a result, healthcare providers and clinicians now face several indirect responsibilities. These include: interpreting system logs; ensuring accurate documentation; maintaining data quality; reporting and supporting incident detection; and enabling traceability through daily system interactions. Such responsibilities require greater digital and regulatory literacy from staff. They can also disrupt workflows, raise questions about the need for dedicated personnel, and increase the operational burden and liability concerns. Similar concerns about complexity under new AI regulations, especially increased compliance duties for healthcare providers as deployers under the AIA, are noted in [13]. To make these duties manageable, departments can build on existing MDR-oriented quality management, risk management, and reporting routines, and extend the responsibilities of teams that already support medical devices to include AIA-related requirements. Reports and escalations can therefore follow established MDR compliance channels, supplemented where needed with any additional documentation or steps required under the AIA. In parallel, effective oversight depends on how daily duties are assigned within the standard of care. In our prostate MRI triage case study, radiologists primarily review non-triaged studies. But, rule-out cases still need a monitoring strategy that does not default to reading all triaged cases, for example, by randomly sampling a fixed proportion. Moreover, oversight activities can be distributed across existing roles in the prostate cancer pathway. Radiologists can conduct periodic sample checks and participate in discrepancy reviews. MRI operators can ensure acquisition quality and flag artifacts or protocol deviations that may affect AI performance. Downstream teams, such as urology and pathology, can provide feedback on clinically significant discordances during routine assessment. To support traceability with minimal extra burden, a light evidence trail can be created during the standard of care pathway by recording structured fields in Radiology Information Systems (RIS) or Picture Archiving and Communication Systems (PACS), such as the AI output, whether it was overridden, and a brief reason.

The regulatory burden associated with AI systems, including continuous documentation updates and renewed conformity assessments, can disrupt clinical workflows and strain resources. As emphasized in [16], stakeholders’ concerns about overlapping requirements from the AIA and MDR underscore the need to streamline regulatory processes. One measure the AIA introduces to address this challenge is the creation of regulatory sandboxes (controlled environments for AI testing) for pre-market testing and validation (Articles 57–59, operational by August 2026). Extending this model into the post-market phase is essential to support safe, efficient system updates throughout the AI lifecycle. Moreover, because horizontal (general-purpose) sandboxes may not fully capture sector-specific constraints, the literature suggests that domain-specific approaches (e.g., healthcare-tailored sandboxes) may offer practical benefits for governing AI in sectoral contexts [30].

This analysis is limited to EU-level obligations under the AIA and MDR, focusing on a prostate MRI triage case study. This narrow scope enabled a deeper, case-specific interpretation of post-market obligations. However, generalizing the findings to other clinical AI applications, such as assistive tools for prostate cancer detection, or to other device risk classes, requires further study. In addition, because this work is anchored in EU-specific regulations, its direct transfer to non-EU regulatory settings is limited. The obligation groups should therefore be read as a structured conceptual framework for comparison rather than as a universally applicable compliance checklist. Our findings should also be considered in light of the evolving regulatory environment, with ongoing amendments and new guidance documents (such as MDCG guidance) that may refine practical expectations over time. Moreover, this is a non-empirical study, and the implications discussed are derived from our legal mapping and case study analysis rather than being directly measured in clinical settings. For future work, we suggest both regulatory-focused case studies and implementation-oriented research to identify domain-specific gaps and to inform adaptable compliance strategies. Future work should also examine how radiology departments can meet deployer obligations sustainably, including which oversight and monitoring tasks can be partly automated without compromising clinical reliability or patient safety. Additionally, more research is needed to translate human oversight requirements into workflow-ready standards. This includes training expectations, escalation rules, and auditing methods that support safe use without eroding the efficiency gains that motivate triage systems.

## Conclusion

This study identifies post-market obligations under the AIA and MDR for a high-risk Class III AI SaMD used for prostate cancer triage in radiology. We extracted and organized these requirements into ten thematic obligation groups and indicated where the two frameworks converge and diverge. Our findings point to expanded post-market responsibilities for healthcare providers and clinicians, including documentation, reporting, and integration of human oversight into routine clinical workflows. However, current ambiguity around human oversight highlights the need for clearer guidance to support safe and efficient clinical use. We therefore call on regulators and standard-setting bodies, in consultation with healthcare providers and manufacturers, to provide more operational guidance on post-market obligations for clinical AI. This especially concerns human oversight, including minimum oversight actions, escalation thresholds, documentation expectations, and division of responsibilities between healthcare providers and manufacturers, ideally aligned across the AIA and MDR. In parallel, radiology departments deploying such systems face a dual challenge of meeting new compliance demands while preserving clinical efficiency, and should therefore prepare proactively. A practical way forward is to integrate oversight, documentation, and escalation pathways into existing quality and patient safety routines rather than treating them as standalone regulatory work. As prostate MRI triage tools move from pilots to routine service, the framework presented here can help departments turn regulatory requirements into clear roles, actionable policies, and workflows.

## Supplementary Information

Below is the link to the electronic supplementary material.

Supplementary Material 1

## Data Availability

No datasets were generated or analysed during the current study.
