# Supplementary material for: The AI Act and the MDR post-market requirements for semiautonomous AI SaMD: a radiology case study in prostate cancer
Source: Abdom Radiol (NY). 2026 Feb 20;51(9):4661–72. doi: 10.1007/s00261-026-05434-z (PMC13476294; doi:10.1007/s00261-026-05434-z)
Supplement: Supplementary file 1 [file 261_2026_5434_MOESM1_ESM.zip › ESM_2.pdf]

Article title: The AI Act and the MDR Post-Market Requirements for Semiautonomous AI SaMD: A Radiology Case Study in Prostate Cancer
Journal name: Abdominal Radiology
Author names: Shakila Shojaei, Derya Yakar, Nynke Vellinga, Vilma Bozgo, Thomas Kwee, Henkjan Huisman, Jeanne Pia Mifsud Bonnici
Corresponding author:
Name: Shakila Shojaei
Affiliation: Department of Radiology, University Medical Center Groningen (UMCG), Groningen, The Netherlands
ORCID: https://orcid.org/0009-0006-8909-8471
Email: s.shojaei@umcg.nl

Supplementary Table 1 Post-market monitoring (PMM) obligations of artificial intelligence (AI) providers and deployers for high-risk AI systems under the EU Artificial Intelligence Act (AIA), applied to an AI-assisted prostate cancer triage case study.

Article Title	Article Number	Obligation	Description	Device	Obligation Holder [a]		Cross-References
					AI Provider	AI Deployer	
Risk management system	9(2)(c)	Continuously Evaluate Emerging Risks Using PMM Data	Providers must continuously evaluate emergent risks, beyond those initially identified in the risk management system, using data obtained from the PMM system mandated under Article 72.	High-Risk AI Systems	X		Article 72
Data and data governance	10(1-5)	Ensure Data Quality and Governance	Where training, validation, or testing datasets are used, they must be governed by practices appropriate to the intended purpose. This includes ensuring relevance, representativeness, completeness, and accuracy; addressing geographical/contextual gaps; detecting and mitigating bias; and applying safeguards when processing special categories of personal data.	High-Risk AI Systems	X		Regulation (EU) 2016/679, Regulation (EU) 2018/1725, Directive (EU) 2016/680
Technical documentation	11(1)	Keep Technical Documentation Continuously Updated	The technical documentation must be kept up to date throughout the system's life cycle.	High-Risk AI Systems	X		N/A

Article Title	Article Number	Obligation	Description	Device	Obligation Holder [a]		Cross-References
					AI Provider	AI Deployer	
Record-keeping	12(1)	Enable Automatic Logging Throughout the System's Lifetime	Providers must ensure that high-risk AI systems support automatic event logging throughout their lifetimes.	High-Risk AI Systems	X		N/A
	12(2)(a)	Log System Events Indicating Risks or Substantial Modifications	Providers must ensure logs capture events that signal potential risks (as defined in Article 79(1)) or substantial system changes. If the EU Medical Device Regulation (MDR) or the EU In Vitro Diagnostic Regulation (IVDR) applies, their rules take precedence over Article 79(1).	High-Risk AI Systems	X		Article 79(1)
	12(2)(b)	Log Events for PMM	Providers must ensure logs support PMM as required under Article 72.	High-Risk AI Systems	X		Article 72
	12(2)(c)	Log Events to Enable Operational Monitoring by Deployers	Providers must ensure logs support deployers' ability to monitor real-world use and detect risks or incidents, as required under Article 26(5).	High-Risk AI Systems	X		Article 26(5)
Human oversight	14(1)	Design and Develop AI Systems for Effective Human Oversight during Use	Providers must design and develop high-risk AI systems that use appropriate human-machine interfaces so that natural persons can effectively oversee the systems during use.	High-Risk AI Systems	X		N/A
	14(2)	Ensure Oversight Reduces Risks	Providers must ensure that the human oversight setup helps prevent or reduce risks to health, safety, or fundamental rights that may arise from intended use or foreseeable misuse, especially when such risks are not already covered by other AI system requirements.	High-Risk AI Systems	X		N/A
	14(3)-1	Align Oversight Measures with Risk, Autonomy, and Context of the System	Providers must ensure that the human oversight measures are proportionate to the system's risk level, its degree of autonomy, and the specific context in which it is used.	High-Risk AI Systems	X		N/A
	14(3)-2	Identify and Implement Human Oversight Measures	Providers must identify human oversight measures before placing the AI system on the market. These may include:<br>a) Measures integrated into the system by the provider, where technically feasible;<br>b) Measures to be implemented by the deployer based on provider instructions.	High-Risk AI Systems	X	*	N/A
	14(4)(a)	Ensure Oversight Personnel Can Understand and Monitor the System	Providers must ensure that the system is delivered to deployers in a way that enables oversight personnel to understand system capabilities and limitations, and to monitor its operation to detect anomalies, failures, or unexpected performance.	High-Risk AI Systems	X		N/A
	14(4)(b)	Ensure Oversight Personnel Are Alert to Automation Bias	Providers must ensure that oversight personnel are aware of the risk of over-reliance on system output, particularly when the system provides recommendations or information.	High-Risk AI Systems	X		N/A

Article Title	Article Number	Obligation	Description	Device	Obligation Holder [a]		Cross-References
					AI Provider	AI Deployer	
	14(4)(c)	Enable Oversight Personnel to Interpret Outputs	Providers must ensure that oversight personnel have the means to correctly interpret system outputs, using available explanation tools or guidance.	High-Risk AI Systems	X		N/A
	14(4)(d)	Ensure Oversight Personnel Can Override or Disregard Output	Providers must ensure that oversight personnel can decide to ignore, override, or reverse the system's output in specific cases.	High-Risk AI Systems	X		N/A
	14(4)(e)	Enable Safe System Interruption by Oversight Personnel	Providers must ensure that oversight personnel can intervene in the system or interrupt it safely (e.g., via a stop button).	High-Risk AI Systems	X		N/A
Accuracy, robustness and cybersecurity	15(1)	Ensure Accuracy, Robustness, Cybersecurity, and Consistent Performance Throughout the System Life Cycle	Providers must design and develop high-risk AI systems to achieve appropriate levels of accuracy, robustness, and cybersecurity, and ensure consistent performance across the system's life cycle.	High-Risk AI Systems	X		N/A
	15(4)	Ensure Feedback Loops Are Not Influenced by Biased Outputs	Providers must ensure that continually learning high-risk AI systems (that continue to learn after being placed on the market or put into service) are designed to prevent biased outputs from affecting future inputs (to next iterations), and must implement safeguards to detect and mitigate harmful feedback loops.	High-Risk AI Systems	X		N/A
	15(5)	Maintain Cybersecurity Against Post-Deployment Attacks	Where appropriate, providers must include measures to prevent, detect, respond to, resolve, and control attacks such as:<br>- Data poisoning (attacks trying to manipulate the training dataset)<br>- Model poisoning (pre-trained components used in training)<br>- Adversarial inputs (inputs designed to cause the AI model to make a mistake)<br>- Confidentiality attacks<br>- Exploitation of model flaws	High-Risk AI Systems	X		N/A
Obligations of providers of high-risk AI systems	16(e)	Keep System Logs While the System Is Under Provider Control	Providers must keep automatically generated logs by their high-risk AI systems, as required under Article 19, even when the system is under their control.	High-Risk AI Systems	X		Article 19
	16(j)	Take Corrective Action and Provide Information to Stakeholders	Providers must take corrective actions and supply information to stakeholders as required by Article 20.	High-Risk AI Systems	X		Article 20
	16(k)	Demonstrate Compliance on Request	Providers must demonstrate compliance with Section 2 of Chapter III requirements when requested by national authorities.	High-Risk AI Systems	X		Chapter III (Section 2)

Article Title	Article Number	Obligation	Description	Device	Obligation Holder [a]		Cross-References
					AI Provider	AI Deployer	
Quality management system	17(1)(a)	Include a Regulatory Compliance Strategy in the Quality Management System (QMS) to Manage Modifications	Providers must include in their QMS a strategy for regulatory compliance, covering how conformity assessments are performed and how system modifications are managed.	High-Risk AI Systems	X		N/A
	17(1)(h)	Integrate the PMM System into the QMS	Providers must include the setup, implementation, and maintenance of a PMM system, as required by Article 72.	High-Risk AI Systems	X		Article 72
	17(1)(I)	Include Procedures for Serious Incident Reporting	Providers must include in the QMS the procedures for reporting serious incidents, in accordance with Article 73.	High-Risk AI Systems	X		Article 73
Documentation keeping	18(1)	Retain Technical Documentation for 10 Years	For 10 years after placing a high-risk AI system on the market or putting it into service, the provider must keep the following documentation available for National Competent Authorities (NCAs): (a) Technical documentation (Article 11), (b) QMS documentation (Article 17), (c) Records of approved changes by notified bodies (if applicable), (d) Decisions and documents issued by notified bodies (if applicable), (e) EU declaration of conformity (Article 47)	High-Risk AI Systems	X		Article 11, Article 17, Article 47
Automatically generated logs	19(1)	Keep Automatically Generated Logs for at Least Six Months	Providers must keep automatically generated logs from their high-risk AI systems (as referred to in Article 12(1)) for at least six months, or longer if appropriate to the system's intended purpose, as long as the logs are under their control and not restricted by Union or national law.	High-Risk AI Systems	X		Article 12(1)
Corrective actions and duty of information	20(1)	Take Corrective Action When Nonconformity Is Identified and Inform Stakeholders	Providers must immediately take corrective actions (such as updating to bring the system into conformity, disabling, withdrawing, or recalling the system) if they consider or have reason to believe that a high-risk AI system they placed on the market or put into service is not compliant with the AIA. They must also inform distributors, and where applicable, deployers, authorized representatives, and importers.	High-Risk AI Systems	X		N/A
	20(2)-1	Investigate Risk in Collaboration with Deployers	Providers must, upon becoming aware of a risk under Article 79(1), immediately investigate the causes of the non-compliance, in collaboration with the reporting deployer if applicable.	High-Risk AI Systems	X	*	Article 79(1)

Article Title	Article Number	Obligation	Description	Device	Obligation Holder [a]		Cross-References
					AI Provider	AI Deployer	
	20(2)-2	Notify Authorities and Certifiers about the Risk	Providers must notify the relevant Market Surveillance Authority (MSA) and, if applicable, the notified body that issued the conformity certificate (in accordance with Article 44), including the nature of the non-compliance and corrective actions taken.	High-Risk AI Systems	X		Article 44
Cooperation with competent authorities	21(1)	Provide Documentation to Competent Authorities	Providers must, upon a reasonable request from a competent authority, supply all necessary information and documentation to demonstrate conformity with the requirements of Chapter III, Section 2, in a clearly understandable EU language designated by the Member State.	High-Risk AI Systems	X		Chapter III (Section 2)
	21(2)	Provide Competent Authorities Access to Logs upon Request	Providers must, upon a reasoned request from a competent authority, grant access to the system logs referred to in Article 12(1), to the extent those logs are under their control.	High-Risk AI Systems	X		Article 12(1)
Obligations of deployers of high-risk AI systems	26(2)	Assign Competent and Supported Oversight Personnel	Deployers must assign human oversight to individuals with appropriate competence, training, authority, and support, ensuring effective monitoring.	High-Risk AI Systems		X	Article 14
	26(4)	Ensure the Input Data Are Relevant and Representative	Deployers who control input data must ensure it is relevant and sufficiently representative for the intended purpose.	High-Risk AI Systems		X	N/A
	26(5)	Monitor System Operation and Report Risks or Incidents	Deployers must continuously monitor the AI system during use, in accordance with the instructions for use. If they suspect the system poses a risk (per Article 79(1)), they must suspend its use and notify the provider or distributor, as well as the competent authority. Serious incidents must be reported to the provider and other relevant actors without delay. (Per Article 74(4), for systems under MDR/IVDR, MDR takes precedence over Article 79(1))	High-Risk AI Systems		X	Articles 72, Article 73, Article 79(1)
	26(6)	Keep System Logs Under Control for at Least Six Months	Deployers must retain automatically generated logs under their control for at least six months (or as otherwise required by law).	High-Risk AI Systems		X	Article 12(1)

Article Title	Article Number	Obligation	Description	Device	Obligation Holder [a]		Cross-References
					AI Provider	AI Deployer	
Post-market monitoring by providers and post-market monitoring plan for high-risk AI systems	72(1)	Establishing an AI-Specific PMM System	Providers must establish and document a PMM system proportionate to the AI system's risk and the nature of the AI technologies.	High-Risk AI Systems	X		N/A
	72(2)	Collect and Analyze PMM Data to Ensure Continuous Compliance with the Requirements of High-Risk AI Systems	Providers must ensure that the PMM system actively and systematically collects, documents, and analyzes relevant performance data (e.g., from deployers and logs) throughout the AI system's lifetime to support continuous compliance with Chapter III, Section 2.<br><br>Where applicable, the system must also analyze interactions with other AI systems. This obligation excludes sensitive operational data from law enforcement deployers.	High-Risk AI Systems	X		Chapter III Section 2 Requirements for High-Risk AI Systems
	72(3)	Developing a Structured PMM Plan for the PMM System	The PMM system must be based on a structured PMM plan, which shall be included in the technical documentation referred to in Annex IV.	High-Risk AI Systems	X		Annex IV
	Annex IV(9) [b]	Include PMM System and PMM Plan in the Technical Documentation	As part of the technical documentation required under Article 11(1) for high-risk AI systems, providers must include a detailed description of the system used to evaluate AI performance in the post-market phase, in accordance with Article 72. This must include the PMM plan referenced in Article 72(3).	High-Risk AI Systems	X		Article 11(1)
	72(4)	Ensure an Equivalent Level of Protection When Integrating the PMM System into Existing Sectoral Monitoring Systems	Providers of high-risk AI systems covered by Union harmonization legislation (Annex I, Section A) may integrate necessary PMM elements (as described in paragraphs 1, 2, and 3) into existing monitoring systems, provided they ensure an equivalent level of protection.	High-Risk AI Systems under Annex I Section A	X		Annex I Section A
Reporting of serious incidents	73(1)	Report Any Serious Incident to MSAs	Providers of high-risk AI systems must report any serious incident to the MSAs of the Member States where the incident occurred.	High-Risk AI Systems	X	*	N/A
	73(2)	Submit Report within 15 Days of Becoming Aware of a Serious Incident	Providers must report a serious incident immediately after establishing a causal link (or reasonable likelihood) between the AI system and the incident.<br><br>Reporting must occur within 15 days of awareness by the provider or deployer, considering the severity of the incident (as specified in the following paragraphs).	High-Risk AI Systems	X	*	N/A
	73(3)	Submit Report within 2 Days for Serious Incidents Involving Infringements to Critical Infrastructure	If the incident involves a widespread infringement or qualifies as a serious incident under Article 3(49)(b), meaning it leads to irreversible disruption of critical infrastructure, reporting must be immediate and occur no later than two days after awareness.	High-Risk AI Systems	X	*	Article 3(49)(b)

Article Title	Article Number	Obligation	Description	Device	Obligation Holder [a]		Cross-References
					AI Provider	AI Deployer	
	73(4)	Submit Report within 10 Days for Serious Incidents Involving Fatalities	If the serious incident results in death, reporting must be immediate or as soon as the provider or deployer has established a causal link or it is suspected, but no later than 10 days after awareness.	High-Risk AI Systems	X	*	N/A
	73(5)	Submit Initial Incomplete Report If Necessary to Meet Deadlines	If timely reporting is difficult, the provider (or, where applicable, the deployer) may submit an initial incomplete report followed by a complete report.	High-Risk AI Systems	X	*	N/A
	73(6)-1	Immediate Investigation and Risk Assessment of Serious Incidents	Providers must promptly investigate the serious incident and the AI system involved after reporting the incident. The investigation must include a risk assessment of the incident.	High-Risk AI Systems	X		N/A
	73(6)-2	Preserve AI System State and Cooperate with Authorities	Providers must cooperate with competent authorities and, where applicable, with notified bodies. They must not alter the AI system in a way that could impact the evaluation of the incident before informing authorities.	High-Risk AI Systems	X		N/A
	73(10)	Report Fundamental Rights Incidents Only to NCAs for AI Systems Covered by the MDR or IVDR	For high-risk AI systems that are themselves devices or safety components of devices under MDR or IVDR, providers must report only serious incidents involving fundamental rights (under Article 3(49)(c)) to the NCA designated by the Member State, rather than to the MSA.	High-Risk AI Systems Covered under MDR/IVDR	X		Article 3(49)(c), Regulation (EU) 2017/745 (MDR), Regulation (EU) 2017/746 (IVDR)
Market surveillance and control of AI systems in the Union market	74(1)	Apply Regulation (EU) 2019/1020 to AI Systems Under This Regulation	All operators must ensure that Regulation (EU) 2019/1020 is applied to AI systems within the scope of the AIA.	All AI Systems	*	*	Regulation (EU) 2019/1020, Article 2(1)
	74(5)-1	Ensure Remote MSAs Access for On-Site Inspections and Physical Checks	Providers and deployers must provide conditions where MSAs can remotely exercise the power to conduct unannounced on-site inspections and physical checks of their AI systems and products, as referred to in article 14(4)(d) of Regulation (EU) 2019/1020.	All AI Systems	X	X	Regulation (EU) 2019/1020 (Article 14(4)(d))
	74(5)-2	Ensure Remote MSAs Access for Sampling and Reverse Engineering	Providers and deployers must provide conditions where MSAs can remotely access the ability to acquire product samples, including using cover identities, for inspection and reverse engineering to identify non-compliance as referred to in article 14(4)(j) of Regulation (EU) 2019/1020.	All AI Systems	X	X	Regulation (EU) 2019/1020 (Article 14(4)(j))
	74(12)	Grant MSAs Access to Documentation and Datasets upon Request	Without prejudice to the powers provided for under Regulation (EU) 2019/1020, MSAs must have full access to documentation and datasets (training, validation, and testing) for high-risk AI systems, as necessary to fulfill their tasks. This access may be granted remotely via Application Programming Interfaces (APIs) or other secure technical means, subject to security safeguards.	High-Risk AI Systems	X		Regulation (EU) 2019/1020

Article Title	Article Number	Obligation	Description	Device	Obligation Holder [a]		Cross-References
					AI Provider	AI Deployer	
	74(13)	Grant MSAs Access to Source Code Only If Necessary and If Other Checks Have Been Exhausted	MSAs must have access to the source code of high-risk AI systems upon a reasoned request only if:<br>(a) This access is necessary to assess compliance with Chapter III, Section 2 requirements; and<br>(b) Other verification methods (e.g., testing, auditing, or data/documentation review) have been exhausted.	High-Risk AI Systems	X		Chapter III Section 2 Requirements for High-Risk AI Systems
**Right to explanation of individual decision-making**	86(1)	Provide Clear Explanations of AI Decisions with Legal or Significant Effects on Individuals' Rights and Safety	Deployers must explain the role of the AI system and the main elements of any decision that has legal or significant effects and adversely impacts the individual's rights and safety, except for systems under Annex III(2).	High-Risk AI Systems (Excluding Annex III(2) Critical Infrastructure)		X	Annex III(2)
	86(2)	Apply Legal Exceptions to Explanation Rights If Union or National Law Limits or Overrides the Right to Explanation	If Union or national law lawfully limits or overrides the right to explanation, deployers must recognize and follow those exceptions.	High-Risk AI Systems (Excluding Annex III(2) Critical Infrastructure)		X	N/A
	86(3)	Avoid Redundancy with Other Union Laws When Providing Explanations of AI Decisions	Deployers must apply Article 86(1) only if the right to explanation is not already provided elsewhere in Union law.	High-Risk AI Systems (Excluding Annex III(2) Critical Infrastructure)		X	N/A

[a] "X" indicates the primary obligation holder; "*" indicates obligations that apply only where applicable (i.e., partially or in specific situations)

[b] Obligations derived from extra articles or annexes
