# Supplementary material for: The AI Act and the MDR post-market requirements for semiautonomous AI SaMD: a radiology case study in prostate cancer
Source: Abdom Radiol (NY). 2026 Feb 20;51(9):4661–72. doi: 10.1007/s00261-026-05434-z (PMC13476294; doi:10.1007/s00261-026-05434-z)
Supplement: Supplementary file 1 [file 261_2026_5434_MOESM1_ESM.zip › ESM_3.pdf]

Article title: The AI Act and the MDR Post-Market Requirements for Semiautonomous AI SaMD: A Radiology Case Study in Prostate Cancer
Journal name: Abdominal Radiology
Author names: Shakila Shojaei, Derya Yakar, Nynke Vellinga, Vilma Bozgo, Thomas Kwee, Henkjan Huisman, Jeanne Pia Mifsud Bonnici
Corresponding author:
Name: Shakila Shojaei
Affiliation: Department of Radiology, University Medical Center Groningen (UMCG), Groningen, The Netherlands
ORCID: https://orcid.org/0009-0006-8909-8471
Email: s.shojaei@umcg.nl

Supplementary Table 2 Post-market surveillance (PMS) obligations of manufacturers and healthcare providers for Class III Software as a Medical Device (SaMD) under the EU Medical Device Regulation (MDR), applied to an artificial intelligence (AI)-assisted prostate cancer triage case study.

Article Title	Article Number	Obligation	Description	Device	Obligation Holder [a]		Cross-References
					Manufacturer	Healthcare Provider	
General obligations of manufacturers	10(4)	Maintain Technical Documentation	Manufacturers of devices other than custom-made devices must draw up and update technical documentation to assess conformity. The technical documentation shall include the elements set out in Annexes II and III.	All Devices Except Custom-Made Devices	X		Annex II, Annex III
	10(8)-1	Retain Compliance Documentation for the Specified Period	Manufacturers must retain key compliance documentation (technical documentation, EU declaration of conformity, and any relevant certificates) for 10 years after the last device is placed on the market.	All Non-Implantable Devices	X		Article 56
	10(8)-2	Ensure Authorities Have Access to Documentation	Upon request, manufacturers must provide full or summary technical documentation to the competent authority. Manufacturers outside the EU must ensure their authorized representative has permanent access to this documentation to fulfil their duties under Article 11(3).	All Devices	X		Article 11(3)
	10(9)(General)	Ensure Conformity of Production	Manufacturers must ensure series production remains compliant with the MDR and promptly address any changes in design, harmonized standards, or common specifications used to declare conformity.	All Devices	X		N/A
	10(9)(a)	Include a Regulatory Compliance Strategy in the Quality Management System (QMS)	The QMS must include conformity assessment procedures and mechanisms to manage device modifications.	All Devices Except Investigational Devices	X		N/A

Article Title	Article Number	Obligation	Description	Device	Obligation Holder [a]		Cross-References
					Manufacturer	Healthcare Provider	
	10(9)(f)	Include Clinical Evaluation and Post-Market Clinical Follow-Up (PMCF) in the QMS	The QMS must address clinical evaluation and PMCF in accordance with the MDR.	All Devices Except Investigational Devices	X		Article 61, Annex XIV
	10(9)(i)	Include PMS in the QMS	The QMS must define and implement PMS procedures in line with Article 83.	All Devices Except Investigational Devices	X		Article 83
	10(9)(k)	Include Vigilance and Field Safety Corrective Action (FSCA) Reporting in the QMS	The QMS must define procedures for reporting serious incidents and implementing FSCAs.	All Devices Except Investigational Devices	X		N/A
	10(9)(l)	Include Corrective and Preventive Actions (CAPAs) Management in the QMS	The QMS must include CAPA procedures and verify their effectiveness.	All Devices Except Investigational Devices	X		N/A
	10(9)(m)	Include Monitoring and Product Improvement in the QMS	The QMS must include processes for monitoring system outputs, analyzing performance data, and implementing improvements.	All Devices Except Investigational Devices	X		N/A
	10(10)	Implement and Maintain a PMS System	Manufacturers must implement and keep up to date a PMS system that complies with Article 83.	All Devices	X		Article 83
	10(12)	Take Corrective Action in Case of Non-Compliance	If a manufacturer identifies or suspects that a device is non-compliant with the MDR, they must immediately take corrective action to bring it into conformity, withdraw it, or recall it. Distributors, authorized representatives, and importers must be informed.<br><br>For serious risks, competent authorities and notified bodies must also be notified without delay.	All Devices	X		Article 56
	10(13)	Establish an Incident and FSCA Reporting System	Manufacturers must have a system in place to record and report serious incidents and FSCAs, in line with the vigilance requirements set out in Articles 87 and 88.	All Devices	X		Article 87, Article 88
	10(14)	Provide Information to and Cooperate with Authorities	Manufacturers must, upon request, provide all information and documentation necessary to demonstrate device conformity, in the language required by the relevant Member State. They must also provide free samples or grant access to the device upon request. Cooperation with competent authorities is required for any corrective action to address device risks.	All Devices	X		N/A
**Post-market surveillance system of the manufacturer**	83(1)	Establish a PMS System Suitable for the Device Type and Class as Part of the QMS	Plan, establish, document, implement, maintain, and update a PMS system for each device in a manner that is proportionate to the risk class and appropriate to the device type. This PMS System must be part of the QMS (as per Article 10(9)).	All Devices	X		10(9)
	83(2)	Continuously Collect, Record, and Analyze Device Data in the PMS System and Act on the Results	1. The PMS system should actively and systematically gather, record, and analyze relevant data.<br>2. The data should cover the quality, performance, and safety of the device throughout its entire lifetime.	All Devices	X		N/A

Article Title	Article Number	Obligation	Description	Device	Obligation Holder [a]		Cross-References
					Manufacturer	Healthcare Provider	
			3. The PMS system should enable drawing necessary conclusions.<br>4. The PMS system should support determining, implementing, and monitoring CAPA.				
	83(3)(a)	Use PMS Data to Update Benefit-Risk Determination and Risk Management	Use the PMS data to update the benefit-risk determination and to improve the risk management as referred to in Annex I Chapter I.	All Devices	X		Annex I Chapter I (Risk Management)
	Annex I Chapter I (3)(e) [b]	Evaluate the Impact of PMS on Risk Management	Manufacturers must evaluate the impact of information from the production phase and, in particular, from the PMS system, on hazards and the frequency of occurrence thereof, on estimates of their associated risks, as well as on the overall risk, benefit-risk ratio, and risk acceptability.	All Devices	X		N/A
	83(3)(b)	Use PMS Data to Update Device Design, Instructions for Use (IFU), and Labeling	Use the PMS data to update the design and manufacturing information, the IFU, and the labelling.	All Devices	X		N/A
	83(3)(c)	Use PMS Data to Update Clinical Evaluation	Use the PMS data to update the clinical evaluation.	All Devices	X		N/A
	83(3)(d)	Use PMS Data to Update the Summary of Safety and Clinical Performance (SSCP)	Use the PMS data to update the summary of safety and clinical performance referred to in Article 32	All Devices	X		Article 32
	83(3)(e)	Use PMS Data to Identify CAPA	Use the PMS data for the identification of needs for CAPA or FSCA.	All Devices	X		N/A
	83(3)(f)	Use PMS Data to Improve Usability, Performance, and Safety	Use the PMS data for the identification of options to improve the usability, performance, and safety of the device.	All Devices	X		N/A
	83(3)(g)	Use PMS Data in the PMS of Other Devices	Use the PMS data when relevant to contribute to the PMS of other devices.	All Devices	X		N/A
	83(3)(h)	Use PMS Data for Trend Detection	Use the PMS data to detect and report trends in accordance with Article 88.	All Devices	X		Article 88
	83(4)-1	Take PMS-Driven CAPA and Notify Authorities and the Notified Body	If PMS identifies a need for CAPA, the manufacturer shall implement those measures without delay. After implementing PMS-driven measures, the manufacturer shall inform the competent authorities, and, where applicable, the notified body, about those actions.	All Devices	X		N/A
	83(4)-2	Report PMS-Triggered Serious Incidents per Article 87	Whenever PMS uncovers a serious incident or leads to an FSCA, the manufacturer shall report it in accordance with Article 87.	All Devices Except Investigational Devices	X		Article 87

Article Title	Article Number	Obligation	Description	Device	Obligation Holder [a]		Cross-References
					Manufacturer	Healthcare Provider	
**Post-market surveillance plan**	84	Build a PMS System Based on a Documented PMS Plan	The PMS system (as referred to in Article 83) shall be based on a PMS Plan that must comply with the technical documentation requirements for PMS set out in Section 1.1 of Annex III.<br><br>For devices other than custom-made devices, the PMS Plan shall also be part of the technical documentation specified in Annex II.	All Devices	X		Article 83, Annex III Section 1.1, Annex II
**Periodic safety update report**	86(1)-1	Prepare a Periodic Safety Update Report (PSUR) for Each Device or Device Category	Manufacturers of Class IIa, IIb, and III devices must prepare a PSUR for each device (or relevant category/group) to monitor safety and performance.<br><br>Purpose of the PSUR:<br>1. Summarize results and conclusions from PMS data analysis (of the PMS plan referred to in Article 84).<br>2. Provide a rationale and description of any preventive and corrective actions.<br><br>Components:<br>(a) the conclusions of the benefit-risk determination;<br>(b) The key findings of the PMCF;<br>(c) Device Usage & Market Data: Volume of sales, estimated user population, and, where possible, usage frequency.	Class IIa, Class IIb, Class III	X		Article 84
	86(1)-2	Update the PSUR Annually and Revise the Technical Documentation Accordingly	1. Update at least annually.<br>2. PSUR must be included in technical documentation as specified in Annexes II and III.	Class IIb, Class III	X		Annex II, Annex III
	86(2)	Submit the PSUR Electronically to the Notified Body	Manufacturers of Class III and implantable devices shall submit PSURs electronically to the notified body responsible for conformity assessment (Article 52).	Class III and Implantable Devices	X		Article 92, Article 52

Article Title	Article Number	Obligation	Description	Device	Obligation Holder [a]		Cross-References
					Manufacturer	Healthcare Provider	
Reporting of serious incidents and field safety corrective actions	87(1)	Report Serious Incidents and FSCAs to Competent Authorities via the Electronic System	1. Manufacturers of devices (excluding investigational devices) must report the following to the competent authorities, as per Articles 92(5) & 92(7):<br>(a) Any serious incident involving devices on the Union market, except expected side effects documented in product information and covered by trend reporting (Article 88).<br>(b) Any FSCA related to devices on the Union market, including FSCAs conducted in third countries if the reason applies to devices in the EU.<br>2. Reports must be submitted via the electronic system (Article 92).	All Devices Except Investigational Devices	X	*	Article 92, Article 88
	87(2-5)	Comply with Severity-Based Reporting Deadlines	Submit reports within the severity-based deadlines set out in Article 87(2-5) once a causal link is considered plausible:<br>- Report every serious incident within 15 days<br>- Report any serious public-health threat within 2 days<br>- Report death or unexpected serious deterioration cases within 10	All Devices Except Investigational Devices	X		N/A
	87(6)	Submit an On-Time Initial Report Even If Details Are Incomplete	If a timely report is required, but complete details are unavailable, the manufacturer may submit an initial report followed by a complete report.	All Devices Except Investigational Devices	X		N/A
	87(7)	Report Uncertain Incidents within the Applicable Timeline	If the manufacturer is uncertain whether an incident is reportable, it must still submit a report within the applicable timeframe (paragraphs 2-5).	All Devices Except Investigational Devices	X		N/A
	87(8)	Report Any FSCA Before Implementation Unless Immediate Action Is Essential	Except for urgent cases requiring immediate action, manufacturers must report FSCAs in advance before implementation.	All Devices Except Investigational Devices	X		N/A
	87(9)	Replace Individual Incident Reports with Agreed Periodic Summary Reports for Similar Incidents	If multiple serious incidents occur with the same device or device type, the root cause is identified, an FSCA is in place, or the incidents are common and well-documented, the manufacturer may submit periodic summary reports instead of individual incident reports.	All Devices Except Investigational Devices	X		Article 89(9), Article 92(8)
	87(11)	Act on Authority-Notified Suspected Incidents	If a competent authority receives a suspected serious incident report (from healthcare professionals, users, or patients), it must inform the manufacturer without delay.<br>- If the incident is (potentially) serious, then submit a full serious incident report under Article 87 (1-5) and carry out all Article 89 follow-up actions.<br>- If the incident is judged non-serious or is an expected side-effect, then send a reasoned explanatory statement.<br>- If the Authority rejects that explanation, then immediately file the full Article 87 (1-5) report and proceed with Article 89 actions.	All Devices Except Investigational Devices	X		Article 88, Article 89

Article Title	Article Number	Obligation	Description	Device	Obligation Holder [a]		Cross-References
					Manufacturer	Healthcare Provider	
Trend reporting	88(1)-1	Detect and Report Statistically Significant Increases in Non-Serious Incidents or Expected Side Effects	Manufacturers must report any statistically significant increase in:<br>- Non-serious incidents, and/or<br>- Expected undesirable side effects<br>When to report: when the increase could significantly affect the benefit-risk analysis (Annex I, Sections 1 & 5) and may lead to unacceptable risks for patients, users, or others.<br>A statistically significant increase must be determined by comparing the observed frequency and/or severity of incidents against the foreseeable (expected) frequency and/or severity for the device (or the relevant device category/group) over a defined period, as specified in the technical documentation and product information.	All Devices Except Investigational Devices	X		Annex I Section 1, Annex I Section 5, Article 92
	88(1)-2	Integrate Trend Monitoring Methodology in the PMS Plan	Manufacturers must specify in the PMS Plan (Article 84):<br>- How to manage reported incidents.<br>- Methodology for detecting statistically significant increases in frequency or severity.<br>- Observation period used for trend monitoring.	All Devices Except Investigational Devices	X		Article 84
	88(2)	Implement Competent Authority-Mandated Measures After Trend Report Review	If a competent authority, after assessing the manufacturer's trend report, directs that measures be taken, the manufacturer must promptly implement those measures in accordance with the MDR to protect public health and patient safety.	All Devices Except Investigational Devices	X		N/A
Analysis of serious incidents and field safety corrective actions	89(1)-1	Initiate Immediate Investigation and Risk Assessment of Serious Incidents	As soon as a serious incident is reported under Article 87(1), manufacturers must immediately investigate the event and the affected devices. This includes performing a documented risk assessment of the incident and evaluating the need for FSCA.	All Devices Except Investigational Devices	X		Article 87(1)
	89(1)-2	Preserve Evidence and Cooperate During Investigation	Manufacturers must not alter the device or batch sample in any way that could compromise future evaluations until the competent authority has been informed. They must fully cooperate with the competent authority or notified body throughout the investigation process.	All Devices Except Investigational Devices	X		N/A
	89(3)	Supply All Documentation the Competent Authority Needs for Its Risk Assessment	For risk assessment, manufacturers must promptly provide complete documentation to the competent authority upon request.	All Devices Except Investigational Devices	X		Annex I
	89(5)	Submission of Final Manufacturer Investigation Report	The manufacturer must submit a final report to the competent authority through the electronic system (Article 92). The report must include:<br>1. Findings and conclusions from the investigation.<br>2. Any corrective actions to be taken, if necessary.	All Devices Except Investigational Devices	X		Article 92

Article Title	Article Number	Obligation	Description	Device	Obligation Holder [a]		Cross-References
					Manufacturer	Healthcare Provider	
	89(8)	Issue and Distribute Field Safety Notice (FSN) to Device Users	Manufacturers must immediately inform device users about FSCA by issuing a clear FSN in the official EU language(s) determined by the Member State where the action occurs.	All Devices Except Investigational Devices	X		Article 92
**Market surveillance activities**	93(3)	Ensure Competent Authority Access to Documentation and the Device for Market Surveillance Conformity Checks	Economic Operators must provide documentation, information, device samples, or access to devices free of charge when requested by competent authorities (if justified).	All Devices	X		N/A
**Evaluation of devices suspected of presenting an unacceptable risk or other non-compliance**	94	Cooperate with Competent Authority Evaluations of Devices Suspected of Unacceptable Risk or Non-Compliance	Competent authorities must evaluate a device if, based on vigilance, market surveillance, or other data, they have reason to believe the device may present an unacceptable risk to health, safety, or public health, or if it does not comply with the MDR requirements.<br><br>The evaluation must cover all relevant MDR requirements related to the risk or non-compliance.<br><br>Relevant economic operators must cooperate with competent authorities throughout the evaluation process.	All Devices	X		N/A

Article Title	Article Number	Obligation	Description	Device	Obligation Holder [a]		Cross-References
					Manufacturer	Healthcare Provider	
Procedure for dealing with devices presenting an unacceptable risk to health and safety	95(1)	Take Corrective Actions for Devices Presenting Unacceptable Risk	If an evaluation under Article 94 confirms that a device presents an unacceptable risk to health, safety, or public health, competent authorities must act without delay. They shall require the manufacturer, its authorized representative, and other economic operators to take corrective action to:<br>1. Bring the device into compliance with the MDR.<br>2. Restrict the device's availability on the market.<br>3. Impose specific conditions on market availability.<br>4. Withdraw or recall the device from the market.<br>These measures must be proportionate to the risk and must be completed within a clearly defined and communicated timeframe.	All Devices Presenting an Unacceptable Risk to Health and Safety	X		Article 94
	95(3)	Ensure Corrective Actions Are Implemented Promptly Across the Entire Union	Economic operators (manufacturers, authorized representatives, and all other relevant economic operators) must ensure that corrective actions are applied Union-wide for all affected devices without delay.	All Devices Presenting an Unacceptable Risk to Health and Safety	X		N/A
Other non-compliance	97(1)	Correct Non-Compliances That Do Not Pose an Unacceptable Risk Upon Competent Authority Instruction	If a device does not comply with the MDR requirements but does not present an unacceptable risk, competent authorities must require the economic operator to correct the non-compliance within a clearly defined, proportionate, and reasonable timeframe.	All Devices Presenting Non-Compliance That Does Not Pose an Unacceptable Risk	X		Article 94
Electronic programmable systems	Annex I (17.1)	Ensure Software Reliability and Fault Tolerance	Devices with electronic programmable systems (including software) must be designed for repeatability, reliability, and performance. In the event of a single fault, risks must be eliminated or reduced.	All Devices with Electronic Programmable Systems or Standalone Software	X		N/A
	Annex I (17.2)	Follow State-of-the-Art Software Development Practices	Software must be developed using a recognized development lifecycle, integrating risk management (including information security), and must be verified and validated.	Software That Are Medical Devices or Integrated into Medical Devices	X		N/A

Article Title	Article Number	Obligation	Description	Device	Obligation Holder [a]		Cross-References
					Manufacturer	Healthcare Provider	
	Annex I (17.4)	Specify IT Environment and Security Requirements	Minimum hardware and network specs, as well as IT security (e.g., protection from unauthorized access), must be clearly defined to ensure the software runs safely.	All Devices with Electronic Programmable Systems or Standalone Software	X		N/A
POST-MARKET CLINICAL FOLLOW-UP	Annex XIV(5)-1	Define PMCF as a Continuous Process	PMCF must be an ongoing activity under the PMS plan. It updates clinical evaluations by proactively collecting and evaluating clinical data from CE-marked devices used as intended to confirm safety, performance, risk acceptability, and detect emerging risks.	All Devices	X		Article 61, Annex XIV(Part A)
	Annex XIV(5)-2	Actively Collect and Evaluate Clinical Data	PMCF must include real-world data collection from CE-marked devices in use to validate safety and performance, ensure continued acceptability of risks, and identify emerging concerns.	All Devices	X		N/A
	Annex XIV(6)	Follow a Documented PMCF Plan	PMCF activities must follow a documented methodology outlined in a formal PMCF plan.	All Devices	X		N/A
	Annex XIV(6.1)	Define PMCF Plan Objectives	The PMCF plan must aim to: (a) confirm long-term performance and safety, (b) monitor known and detect unknown side effects and risks, (c) verify continued benefit-risk acceptability, and (d) detect misuse or off-label use.	All Devices	X		Annex I(1), Annex I(9)
	Annex XIV(6.2)	Include Required Elements in the PMCF Plan	The PMCF plan must include: (a) general/specific data collection methods (e.g., user feedback, registries), (b) justifications, (c) links to clinical evaluation and risk docs, (d) defined objectives, (e) timelines, and (f) reference to standards/guidance.	All Devices	X		Annex I(3), Annex XIV(Part A)(4)
	Annex XIV(7)	Evaluate and Document PMCF Findings	PMCF results must be analyzed and reported in a PMCF evaluation report, which becomes part of the technical documentation and clinical evaluation.	All Devices	X		N/A

Article Title	Article Number	Obligation	Description	Device	Obligation Holder [a]		Cross-References
					Manufacturer	Healthcare Provider	
	Annex XIV(8)	Use PMCF Results to Update Compliance Documentation	PMCF results must feed back into the clinical evaluation and risk management (Annex I(3)). If PMCF reveals the need for corrective or preventive action, manufacturers must implement it.	All Devices	X		Annex I(3), Article 61, Annex XIV(Part A)

[a] "X" indicates the primary obligation holder; "*" indicates obligations that apply only where applicable (i.e., partially or in specific situations)

[b] Obligations derived from extra articles or annexes
