# Supplementary material for: The AI Act and the MDR post-market requirements for semiautonomous AI SaMD: a radiology case study in prostate cancer
Source: Abdom Radiol (NY). 2026 Feb 20;51(9):4661–72. doi: 10.1007/s00261-026-05434-z (PMC13476294; doi:10.1007/s00261-026-05434-z)
Supplement: Supplementary file 1 [file 261_2026_5434_MOESM1_ESM.zip › ESM_4.pdf]

Article title: The AI Act and the MDR Post-Market Requirements for Semiautonomous AI SaMD: A Radiology Case Study in Prostate Cancer
Journal name: Abdominal Radiology
Author names: Shakila Shojaei, Derya Yakar, Nynke Vellinga, Vilma Bozgo, Thomas Kwee, Henkjan Huisman, Jeanne Pia Mifsud Bonnici
Corresponding author:
Name: Shakila Shojaei
Affiliation: Department of Radiology, University Medical Center Groningen (UMCG), Groningen, The Netherlands
ORCID: https://orcid.org/0009-0006-8909-8471
Email: s.shojaei@umcg.nl

Supplementary Table 3 List of articles from the EU Artificial Intelligence Act (AIA) and EU Medical Device Regulation (MDR)that were excluded from the final analysis.

Exclusion Reason	AIA	MDR
Contains general compliance provisions or lacks explicit post-market duties.	8, 9(1), 9(2)(a-b), 9(2)(d), 9(3-10), 11(2-3), 13, 15(3), 16(a-d), 16(f-i), 16(l), 17(1)(b-g), 17(1)(j-m), 17(2-3), 26(1), 26(3), 26(7-12), 27, 74(4), 74(9), 76	10(1-3), 10(5-7), 10(9)(b-f), 10(9)(g-h), 10(9)(j), 10(11), 10(15-16), Annex I (17.3),
Not applicable to the targeted device.	10(6), 12(3), 14(5), 53-55, 73(9), 74(6-8), 75, 77(1),77(3), 88-94	85, 86(3), 79-83
Not applicable to the targeted obligation holders.	15(2), 17(4), 18(2-3), 19(2), 21(3), 22-25, 73(7), 73(8), 73(11), 74(2-3), 74(10-11), 74(14), 77(2), 77(4), 78, 84-85, 87	87(10), 89(2), 89(4), 89(6-7), 89(9-11), 90-92, 93(1-2), 93(4-11), 95(2), 95(4-7), 96, 97(2-3), 98-100
