# Supplementary material for: The AI Act and the MDR post-market requirements for semiautonomous AI SaMD: a radiology case study in prostate cancer
Source: Abdom Radiol (NY). 2026 Feb 20;51(9):4661–72. doi: 10.1007/s00261-026-05434-z (PMC13476294; doi:10.1007/s00261-026-05434-z)
Supplement: Supplementary file 1 [file 261_2026_5434_MOESM1_ESM.zip › ESM_5.pdf]

Article title: The AI Act and the MDR Post-Market Requirements for Semiautonomous AI SaMD: A Radiology Case Study in Prostate Cancer
Journal name: Abdominal Radiology
Author names: Shakila Shojaei, Derya Yakar, Nynke Vellinga, Vilma Bozgo, Thomas Kwee, Henkjan Huisman, Jeanne Pia Mifsud Bonnici
Corresponding author:
Name: Shakila Shojaei
Affiliation: Department of Radiology, University Medical Center Groningen (UMCG), Groningen, The Netherlands
ORCID: https://orcid.org/0009-0006-8909-8471
Email: s.shojaei@umcg.nl

Supplementary Table 4 Thematic groups of extracted post-market obligations for target obligation holders under the EU Artificial Intelligence Act (AIA) and the EU Medical Device Regulation (MDR), applied to an artificial intelligence (AI)-assisted prostate cancer triage case study representing a Class III high-risk AI Software as a Medical Device (SaMD).

Obligation Group	Obligation	Articles		Device		Obligation Holder [a]			
		AIA	MDR	AIA	MDR	AI Provider (AIA)	AI Deployer (AIA)	Manufacturer (MDR)	Healthcare Provider or Organization (MDR)
1. Planning a post-market oversight system	Embedding a Post-Market Monitoring (PMM)/Post-Market Surveillance (PMS) System within the Quality Management System (QMS)	72(1), 17(1)(h)	83(1), 10(9)(i), 10(9)(f), 10(10)	High-Risk AI Systems	All Devices	X		X	
	Post-Market Data Collection and Use	72(2)	83(2), Annex XIV(5)	High-Risk AI Systems	All Devices	X		X	
	Structured Use of PMM/PMS Data for Continuous Improvement	9(2)(c)	83(3)(a-h), Annex I Chapter I(3)(e), 10(9)(m)	High-Risk AI Systems	All Devices	X		X	
	Structured and Documented PMM/PMS Plan	Article 72(3), Annex IV(9)	Article 84, Annex XIV(6), Annex XIV(6.1), Annex XIV(6.2), Annex XIV(7), Annex XIV(8)	High-Risk AI Systems	All Devices	X		X	
	Alignment of PMM with Existing Sectoral Monitoring Systems	Article 72(4)	N/A	High-Risk AI Systems under Annex I Section A	N/A	X			

Obligation Group	Obligation	Articles		Device		Obligation Holder [a]			
		AIA	MDR	AIA	MDR	AI Provider (AIA)	AI Deployer (AIA)	Manufacturer (MDR)	Healthcare Provider or Organization (MDR)
2. Trend reporting of non-serious incidents	Detecting and Reporting Statistically Significant Increases in Non-Serious Incidents or Expected Side Effects	N/A	Article 88(1)-1	N/A	All Devices Except Investigational Devices			X	
	Integrating Trend Monitoring Methodology in the PMS Plan	N/A	Article 88(1)-2	N/A	All Devices Except Investigational Devices			X	
	Implementing Competent Authority-Mandated Measures after Trend Report Review	N/A	Article 88(2)	N/A	All Devices Except Investigational Devices			X	
3. Serious incident reporting and advance notification of corrective actions	Reporting Serious Incidents and Field Safety Corrective Actions (FSCAs) to Authorities	Article 73(1), Article 73(10), Artcile 17(1)(i), Article 26(6)	Article 87(1), Article 87(8), Article 10(9)(k), Article 10(13)	High-Risk AI Systems	All Devices Except Investigational Devices	X	*	X	*
	Ensuring Reporting within Severity-Based Timeframes	Article 73(2–4)	Article 87(2–5)	High-Risk AI Systems	All Devices Except Investigational Devices	X	*	X	
	Submitting Incomplete Reports When Necessary	Article 73(5)	Article 87(6)	High-Risk AI Systems	All Devices Except Investigational Devices	X	*	X	
	Reporting Uncertain Incidents within the Applicable Timeline	N/A	Article 87(7)	N/A	All Devices Except Investigational Devices			X	
	Replacing Individual Incident Reports with Periodic Summaries	N/A	Article 87(9)	N/A	All Devices Except Investigational Devices			X	
	Reporting Authority-Triggered Serious Incidents	N/A	Article 87(11)	N/A	All Devices Except Investigational Devices			X	
	Reporting PMS-Triggered Serious Incidents	N/A	83(4)-2	N/A	All Devices Except Investigational Devices			X	

Obligation Group	Obligation	Articles		Device		Obligation Holder [a]			
		AIA	MDR	AIA	MDR	AI Provider (AIA)	AI Deployer (AIA)	Manufacturer (MDR)	Healthcare Provider or Organization (MDR)
4. Investigation and risk evaluation after serious incident reporting	Initiating Investigation and Risk Assessment of Serious Incidents	Article 73(6)-1, Article 20(2)-1	Article 89(1)-1	High-Risk AI Systems	All Devices Except Investigational Devices	X	*	X	
	Preserving System State and Cooperating During Investigations	Article 73(6)-2	Article 89(1)-2	High-Risk AI Systems	All Devices Except Investigational Devices	X		X	
	Providing Documentation to Authorities for Post-Incident Assessment	Article 21(1)	Article 89(3)	High-Risk AI Systems	All Devices Except Investigational Devices	X		X	
	Submitting the Final Investigation Report to Authorities	Article 20(2)-2	Article 89(5)	High-Risk AI Systems	All Devices Except Investigational Devices	X		X	
5. Corrective actions and safety notices after serious incidents	Issuing and Distributing Field Safety Notices (FSNs) to Device Users	N/A	Article 89(8)	N/A	All Devices Except Investigational Devices			X	
	Implementing Corrective and Preventive Actions (CAPA) and Informing Authorities and Stakeholders	Article 20(1), Article 16(j), Article 20(2)-2	Article 83(4)-1, Article 10(12), Article 10(9)(l)	High-Risk AI Systems	All Devices	X		X	

Obligation Group	Obligation	Articles		Device		Obligation Holder [a]			
		AIA	MDR	AIA	MDR	AI Provider (AIA)	AI Deployer (AIA)	Manufacturer (MDR)	Healthcare Provider or Organization (MDR)
6. Addressing suspected risks and non-compliance outside incident reporting pathways	Cooperating with the Competent Authority in Evaluations of Devices Suspected of Unacceptable Risk or Non-Compliance	Article 21	Article 94	High-Risk AI Systems	All Devices	X		X	
	Taking Corrective Actions for Devices Presenting an Unacceptable Risk	Article 20(1), Article 16(j)	Article 95(1), Article 95(3), Article 10(12)	High-Risk AI Systems	All Devices Presenting an Unacceptable Risk to Health and Safety	X		X	
	Correcting Non-Compliance That Does Not Pose an Unacceptable Risk	N/A	Article 97(1)	N/A	All Devices Presenting Non-Compliance That Does Not Pose an Unacceptable Risk			X	
	Demonstrating Compliance upon Request	Article 16(k)	Article 10(9)(General)	High-Risk AI Systems	All Devices	X		X	
	Including a Regulatory Compliance Strategy in the QMS to Manage Modifications	Article 17(1)(a)	Article 10(9)(a)	High-Risk AI Systems	All Devices Except Investigational Devices	X		X	
7. Market surveillance readiness across the product life cycle	Structured Safety Reporting Using the Periodic Safety Update Report (PSUR)	N/A	Article 86	N/A	Class IIa, IIb, III, and Implantable Devices			X	
	Applying Regulation (EU) 2019/1020	Article 74(1)	Applying Where Applicable under Regulation (EU) 2019/1020 According to Its Scope (Article 2)	All AI Systems	All Devices	*	*	*	*
	Ensuring Access for Competent Authorities and Market Surveillance Authorities (MSAs) to Documentation, the Device, Code, Logs, and Datasets for Market Surveillance Conformity Checks	Article 74(5), Article 74(12), Article 74(13), Article 21(1), Article 21(2)	Article 93(3), Article 10(8)-2, Article 10(14)	High-Risk AI Systems (74(5)), All AI Systems (74(12), 74(13), 21(1), 21(2))	All Devices	*	*	X	

Obligation Group	Obligation	Articles		Device		Obligation Holder [a]			
		AIA	MDR	AIA	MDR	AI Provider (AIA*)*	AI Deployer (AIA)	Manufacturer (MDR)	Healthcare Provider or Organization (MDR)
8. Maintaining ongoing documentation and logs	Maintaining Technical Documentation	11(1)	10(4)	High-Risk AI Systems	All Devices Except Custom-Made Devices	X		X	
	Retaining Compliance Documentation for the Specified Period	18(1)	10(8)-1, 10(8)-2	High-Risk AI Systems	All Non-Implantable Devices	X		X	
	Keeping System Logs While the System Is Under Control	16(e), 19(1), 26(6)	N/A	High-Risk AI Systems	N/A	X	X		
	Ensuring Automatic Logging of Events	12(1)	N/A	High-Risk AI Systems	N/A	X			
	Logging Risk-Related Events and Substantial Changes	12(2)(a)	N/A	High-Risk AI Systems	N/A	X			
	Logging Events for Post-Market Monitoring	12(2)(b)	N/A	High-Risk AI Systems	N/A	X			
	Logging Events for Operational Monitoring by Deployers	12(2)(c)	N/A	High-Risk AI Systems	N/A	X			
9. Human oversight	Designing and Developing AI Systems to Enable Effective Human Oversight During Use	Article 14(1)	N/A	High-Risk AI Systems	N/A	X			
	Ensuring Oversight Reduces Risks	Article 14(2)	N/A	High-Risk AI Systems	N/A	X			
	Aligning Oversight Measures with System Risk, Autonomy, and Context	Article 14(3)-1	N/A	High-Risk AI Systems	N/A	X			
	Identifying and Implementing Human Oversight Measures	Article 14(3)-2	N/A	High-Risk AI Systems	N/A	X	*		
	Ensuring Oversight Personnel Can Understand, Interpret, Override, and Interrupt the System	Article 14(4)	N/A (partially Annex I, 17(1))	High-Risk AI Systems	N/A	X			
	Assigning Competent and Supported Oversight Personnel	26(2)	N/A	High-Risk AI Systems	N/A		X		
	Monitoring System Operation and Reporting Risks or Incidents	26(5)	N/A	High-Risk AI Systems	N/A		X		

Obligation Group	Obligation	Articles		Device		Obligation Holder [a]			
		**AIA**	**MDR**	**AIA**	**MDR**	**AI Provider (AIA)**	**AI Deployer (AIA)**	**Manufacturer (MDR)**	**Healthcare Provider or Organization (MDR)**
10. Requirements for safe and transparent AI technology	Providing Clear Explanations of AI-Based Decisions	Article 86	N/A	High-Risk AI Systems (Excluding Annex III(2) Critical Infrastructure)	N/A		X		
	Ensuring Accuracy, Robustness, Cybersecurity, and Consistent Performance Throughout the System Lifecycle	15(1)	Annex I (17.1), Annex I (17.2), Annex I (17.4)	High-Risk AI Systems	All Devices with Electronic Programmable Systems or Standalone Software	X		X	
	Ensuring Feedback Loops Are Not Influenced by Biased Outputs	15(4)	N/A	High-Risk AI Systems	N/A	X			
	Maintaining Cybersecurity Against Post-Deployment Attacks	15(5)	Annex I (17.4)	High-Risk AI Systems	All Devices with Electronic Programmable Systems or Standalone Software	X		X	
	Ensuring Input Data Are Relevant and Representative	26(4)	N/A	High-Risk AI Systems	N/A		X		
	Ensuring Data Quality and Governance	10(1-5)	N/A	High-Risk AI Systems	N/A	X			

[a] "X" indicates the primary obligation holder; "*" indicates obligations that apply only where applicable (i.e., partially or in specific situations)
