# Supplementary material for: The AI Act and the MDR post-market requirements for semiautonomous AI SaMD: a radiology case study in prostate cancer
Source: Abdom Radiol (NY). 2026 Feb 20;51(9):4661–72. doi: 10.1007/s00261-026-05434-z (PMC13476294; doi:10.1007/s00261-026-05434-z)
Supplement: Supplementary file 1 [file 261_2026_5434_MOESM1_ESM.zip › ESM_1.pdf]

Article title: The AI Act and the MDR Post-Market Requirements for Semiautonomous AI SaMD: A Radiology Case Study in Prostate Cancer
Journal name: Abdominal Radiology
Author names: Shakila Shojaei, Derya Yakar, Nynke Vellinga, Vilma Bozgo, Thomas Kwee, Henkjan Huisman, Jeanne Pia Mifsud Bonnici
Corresponding author:
Name: Shakila Shojaei
Affiliation: Department of Radiology, University Medical Center Groningen (UMCG), Groningen, The Netherlands
ORCID: https://orcid.org/0009-0006-8909-8471
Email: s.shojaei@umcg.nl

Supplementary Text 1 Regulatory Applicability and Classification of the Case Study

The artificial intelligence (AI) Software as a Medical Device (SaMD) analyzed in this study is a machine-based system that processes medical data to generate diagnostic outputs, such as identifying cases of clinically significant prostate cancer (csPCa). It operates with a degree of autonomy by excluding high-certainty normal cases from radiologist review, thereby influencing clinical decision-making. Accordingly, it meets the definition of an AI system under Article 3(1) of the EU Artificial Intelligence Act (AIA). At the same time, it qualifies as a medical device under Article 2(1) of the EU Medical Device Regulation (MDR) because it is designed to support the diagnosis of prostate cancer. Since its main functionality (analyzing medical data) is carried out entirely through software, and not through any pharmacological, immunological, or metabolic action, the system is not excluded from the MDR.

In our analysis, we begin classification with the MDR, as the AIA determines risk level partly on the basis of a device's classification under sectoral laws such as the MDR. Under the MDR, device class is determined by intended use and risk level, as outlined in Annex VIII. The AI system in our case study qualifies as an active medical device, since it uses software to perform diagnostic functions without direct human input. This brings it under the classification rules for active devices, particularly Rules 9-13. Among these, Rule 11 is the most relevant, as it applies to software intended to provide information used for diagnostic or therapeutic decision-making. By default, such software is classified as Class IIa. If the decision could result in serious deterioration of health or require surgical intervention, it is classified as Class IIb. If incorrect outputs could lead to death or irreversible harm, the system must be classified as Class III. In our case study, the AI system is designed to automatically exclude certain patients from radiologist review. Missing a clinically significant cancer could delay diagnosis and treatment, potentially causing serious or life-threatening outcomes. Therefore, based on the potential risk level and the strictest interpretation of Rule 11, we classify the system as Class III. For further guidance, pages 17-25 of the Medical Device Coordination Group (MDCG) 2021-24 document [1] provide a visual summary of the classification rules.

Under the AIA, our system is not prohibited because none of the conditions listed in Article 5 apply. It qualifies as a high-risk AI system under Article 6(1), since it is a medical device covered by the MDR (listed in Annex I of AIA) and requires third-party conformity assessment due to its Class III classification.

Supplementary References

[1] Medical Device Coordination Group (MDCG) (2021) MDCG 2021-24 Guidance on classification of medical devices. Medical Device Coordination Group. https://health.ec.europa.eu/system/files/2021-10/mdcg_2021-24_en_0.pdf. Accessed 31 Dec 2025
